# Quantized neural representation for lossy cryo-EM compression

**DOI:** 10.1093/bioinformatics/btag547

**Published:** 2026-07-23

**Authors:** Xi Duan, Yiming Shao, ZhiYuan Meng, Zijian Xu, Jun Han, Changhe Tu, Yunhai Wang, Renmin Han, Qiong Zeng

**Affiliations:** Interdisciplinary Center, Shandong University, Jinan 250100, China; School of Computer Science and Technology, Shandong University, Qingdao 266237, China; School of Computer Science and Technology, Shandong University, Qingdao 266237, China; School of Computer Science and Technology, Shandong University, Qingdao 266237, China; Netease Games, Hangzhou, Zhejiang 310052, China; Division of Emerging Interdisciplinary Areas, the Hong Kong University of Science and Technology, Hong Kong, China; School of Computer Science and Technology, Shandong University, Qingdao 266237, China; School of Information, Renmin University of China, Beijing 100872, China; Research Center for Mathematics and Interdisciplinary Sciences, Shandong University, Qingdao 266000, China; School of Computer Science and Technology, Shandong University, Qingdao 266237, China

## Abstract

**Summary:**

Cryo-electron microscopy (cryo-EM) visualization transforms high-resolution 3D density volumes into intuitive representations, playing a vital role in structural biology. However, the increasing scale of cryo-EM data poses challenges for interactive visualization, including storage, transmission, and exploration. To address this, we propose a hybrid quantized implicit neural representation (INR) method that compresses cryo-EM volumes while supporting efficient on-demand access. To evaluate its effectiveness, we benchmark our approach against traditional compression techniques and one classic INR compressor, assessing both compression efficiency and visual quality. Beyond standard metrics, we examine performance on key cryo-EM tasks, including overall structure identification, secondary structure recognition, and fine-chain inspection. Our results demonstrate that the quantized INR achieves superior storage efficiency and task-relevant fidelity, and we provide an interactive tool and guidelines to assist users in selecting optimal compression strategies.

**Availability:**

To facilitate future research, we provide our quantized neural representation approach and interactive tool available at Zenodo (https://doi.org/10.5281/zenodo.19688284) and GitHub (https://github.com/ChiefMoo/Lossy-Cryo-EM-Compression)

## 1 Introduction

**Figure 1:**
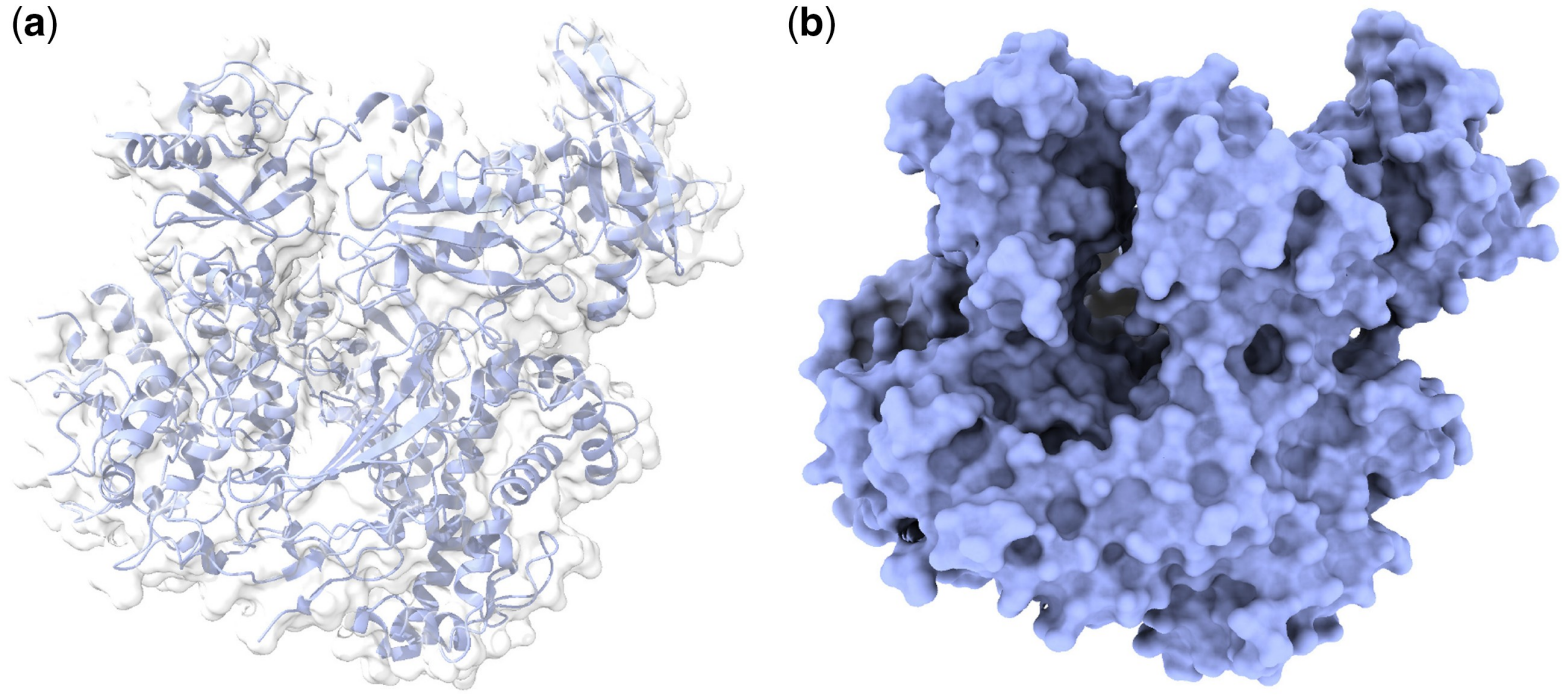
Examples of two cryo-EM visualization techniques. (a) By applying a hybrid of indirect and direct volume rendering, users can compare density distributions around the fitted structures. (b) Users can apply iso-surface rendering to examine the relationship between structures and their functions.

Cryo-electron microscopy (cryo-EM) enables high-resolution 3D visualization of macromolecular structures, offering near-atomic resolution through intuitive density map representations. These 3D density maps are commonly used in cryo-EM visualization, storing volumetric data reconstructed from cryo-EM images. For example, as shown in Fig. 1a, users can compare density distributions and fitted protein structures using a hybrid approach that combines indirect and direct volume rendering (Martinez *et al*. 2019). Alternatively, they can explore the relationship between protein structures and their functions by analyzing volume iso-surface (Humphrey *et al*. 1996, Rodríguez-Guerra Pedregal and Maréchal 2018, Sehnal *et al*. 2021), as shown in Fig. 1b. This approach is central to structural biology, for example, in determining essential secondary structure elements (Pettersen *et al*. 2021). However, as the size and quantity of cryo-EM density maps continue to increase, challenges in interactive visualization arise, particularly regarding data transmission, storage, and exploration (Rose and Hildebrand 2015). These issues complicate efficient cryo-EM data visualization and analysis.

Lossy compression provides a promising approach to the growing scale of cryo-EM data. For example, Mol* (Sehnal *et al*. 2021) applies quantization and down-sampling (Sehnal *et al*. 2020) to balance data reduction, efficiency, and noise sensitivity. Other 3D volume compression methods, such as transform-based techniques and predictive coding (Millidge *et al*. 2022), reduce storage and transmission costs but typically require full decompression for visualization, increasing rendering overhead. In contrast, implicit neural representation (INR) methods (Devkota and Pattanaik 2023, Wu *et al*. 2024) enable large-scale interactive visualization by decompressing only view-dependent coordinates. Nevertheless, the low signal-to-noise ratios, varying resolutions, large data sizes, and complex analytical tasks associated with cryo-EM density maps (Rose and Hildebrand 2015) pose significant challenges. Overall, traditional methods offer fast decompression but limited compression ratios and on-demand access, whereas INR-based approaches provide greater flexibility at higher computational and memory cost.

In this study, we propose a quantized implicit neural representation (INR) method for compressing cryo-EM volumes, enabling high compression ratios while supporting efficient on-demand access for visualization. Compared with existing INR-based compression methods, our method explicitly incorporates feature quantization into the INR framework, aiming to improve storage efficiency while preserving visual fidelity for cryo-EM visualization. Our approach encodes density maps using a multi-level hash grid-based multilayer perceptron with hash-table quantization. To mitigate quantization artifacts, we introduce uniform noise during training to better align learned features with the quantized representation. This framework enables compact storage and fast decompression.

To evaluate effectiveness, we compare our method with traditional compression techniques and unquantized INR approaches using 80 randomly selected cryo-EM density maps from EMDB (wwPDB Consortium 2023). All datasets exceed 100 MB and span resolutions from below 3.5 Å to above 8 Å (Å (Ångström) is a unit of length used in cryo-EM to represent the smallest resolvable distance between two points, where 1 Å=0.1 nm), covering diverse structural characteristics. Compression quality is measured with SSIM and PSNR for overall fidelity and FSC for structural preservation. We also conduct a qualitative study across five representative cryo-EM analytical tasks, including low-resolution structure identification, medium-resolution secondary structure recognition, and high-resolution chain inspection. To better reflect structural biology workflows, we focus on iso-surface visualization rather than full volumes, reducing the influence of internal density variations (Pettersen *et al*. 2021).

Our evaluation shows that while traditional compression methods achieve fast decompression, they struggle at high compression ratios and do not support coordinate-level on-demand access, increasing memory demands during visualization (Wu *et al*. 2024). INR-based techniques, including our quantized INR, maintain more stable visual quality under high compression ratios and provide flexible access. Compared to existing methods, ours achieves superior visual fidelity, efficient decompression times comparable to traditional methods, and effective support for interactive visualization. Our contributions are as follows:

- A novel quantized neural representation approach that optimizes visual quality and computational efficiency in interactive cryo-EM visualization while supporting on-demand data decompression;
- The first evaluation of both traditional and INR-based compression methods, covering five resolution levels along with analytical tasks, based on three compression metrics and a user study;
- Comprehensive experiments evaluating the performance of traditional and INR-based compression methods, showing the superiority of INR-based methods at high compression ratios;
- An interactive tool for exploring our evaluation dataset, evaluation metrics, and analytical tasks, along with four actionable guidelines to help users select appropriate compression techniques.

## 2 Related work

In this section, we review traditional and deep learning–based lossy data compression methods, with an emphasis on 3D volume data and cryo-EM visualization systems.

### 2.1 Traditional lossy data compression

Numerous lossy compression methods have been developed in image processing, with JPEG (Wallace 1992) widely applied across domains. Here, we focus on two main categories for 3D volumes: quantization and error-bounded compression.

#### 2.1.1 Quantization

Quantization is a technique that maps continuous values to a finite set of discrete levels. For example, Ning and Hesselink (1992) initially applied vector quantization techniques to volume rendering to achieve faster rendering speeds and mitigate data storage and transmission challenges for large datasets. Wei and Levoy (2000) applied the tree-structured vector quantization technique in texture synthesis task, obtaining both effectiveness and capability of generating high-quality results. Meng *et al*. (2010) used the predictive vector quantization technique in handling the transmission and storage of large amounts of triangle and vertex geometry data for rendering geometrically detailed 3D meshes. However, quantization methods for volume data often introduce discretization artifacts, which can degrade visual quality and obscure fine details in high-precision scientific applications.

#### 2.1.2 Error-bounded compression

Error-bounded compression reduces scientific data size while keeping reconstruction error within a user-defined bound, making it widely used in high-performance computing (Saudagar 2022). Yeo and Liu (1995) extended the discrete cosine transform to 3D volumes, enabling on-demand decompression with reduced storage and computation. Lindstrom (2014) introduced ZFP, a fixed-rate, near-lossless scheme that assigns a fixed bit budget per block. Di and Cappello (2016) developed SZ, which applies prediction coding to lower storage costs. Ballester-Ripoll and Pajarola (2016) proposed Tthresh, which uses SVD for smooth compression in higher dimensions. However, traditional methods often struggle to balance high compression ratios with visual quality and usually require full decompression before visualization, limiting scalability. In practice, Eng *et al*. (2019) tested several lossy methods for cryo-EM data, finding suboptimal quality but noting potential for preserving images as historical records.

To preserve analytical fidelity, topology-preserving methods integrate structural constraints into compression. Yan *et al*. (2024) proposed TopoSZ, which maintains critical points and contour-tree features within error bounds. Gorski *et al*. (2025) introduced a framework to enhance compressors with topological control, and Li *et al*. (2025) developed a workflow that preserves Morse-Smale complex topology by correcting extrema and integral lines. These methods are highly relevant to topology preservation, but optimization objectives differ from the visualization-oriented criteria emphasized in our evaluation, including compression ratio, visual fidelity, and on-demand access. This mismatch is further illustrated by a failure case on noisy cryo-EM data, where a topology-preserving compressor shows limited compression gain and high runtime cost, with bridge-like visual artifacts; see the supplementary material, available as supplementary data at *Bioinformatics* online.

### 2.2 Learning-based lossy data compression

#### 2.2.1 Autoencoder-based techniques

Autoencoder-based techniques extend traditional lossy compression by replacing conventional codecs with learnable nonlinear ones, while retaining processes such as quantization and entropy encoding. Ballé *et al*. (2017) introduced a framework using autoencoders and outperformed JPEG/JPEG2000 in visual quality, later enhanced with hierarchical entropy models (Minnen *et al*. 2018) and hyper-priors (Ballé *et al*. 2018) to reduce redundancy. Liu *et al*. (2021) applied autoencoders to volumetric data, achieving higher compression ratios than SZ (Di and Cappello 2016) and ZFP (Lindstrom 2014). However, these methods often assume similar training and test distributions, and lack dynamic bitrate adaptation–a feature supported by traditional codecs for real-time bandwidth constraints.

#### 2.2.2 INR-based techniques

INRs model data as continuous functions from coordinates (e.g. spatial positions) to signal values (e.g. density), enabling on-demand visualization. Lu *et al*. (2021) first used MLPs for volumetric scalar-field compression, and Devkota and Pattanaik (2023) later combined MLPs with multi-resolution hash encoding to capture high-frequency features and improve compression ratios. Subsequent work improved efficiency and scalability through macro-cell acceleration and out-of-core training (Wu *et al*. 2023), hash-grid storage for sparse volumes (Kim *et al*. 2024), time-varying volume representations (Han and Wang 2023), uncertainty-aware scientific visualization (Saklani *et al*. 2024), distributed in-situ visualization with temporal abstraction and caching (Wu *et al*. 2024), and mixture-of-experts modeling for complex time-varying volumes (Han *et al*. 2025).

Despite their advantages, INR-based methods suffer from high time complexity, as each coordinate must be processed by a full MLP, and at high compression ratios, limited parameters often lead to the loss of high-frequency details. In cryo-EM visualization, Ranno and Si (2021) proposed Neural Cryo-EM Maps, an INR-based continuous density representation enabling graph-based protein structure interpretation. Liu *et al*. (2023) introduced CryoFormer, a transformer-based neural representation that better captures local flexibility in biomolecular motion reconstruction, outperforming Lu *et al.*’s method. However, Yang *et al*. (2022) reported that INRs can introduce significant artifacts in complex biomedical data, motivating a further investigation of INR performance on cryo-EM density maps.

### 2.3 Cryo-EM data visualization

Cryo-EM visualization systems have widely supported the analysis of biological structures and molecular dynamics. Early examples include VMD (Humphrey *et al*. 1996), which provides multiple rendering modes (such as backbone tubes, ribbons, and cartoons), and Chimera (Pettersen *et al*. 2004), which popularized isosurface and volume rendering. ChimeraX (Pettersen *et al*. 2021) later extended these capabilities with a modern graphics engine and improved data processing, while Maria *et al*. (2025) introduced VTX, an open-source system using impostor-based and adaptive level-of-detail rendering to improve scalability in real-time molecular visualization. However, most of these systems remain locally deployed, limiting cross-platform data sharing and web-based visualization, where simultaneous data processing and rendering are essential.

To address this, several web-based systems have emerged. NGL Viewer (Rose and Hildebrand 2015) supports online visualization of large macromolecular assemblies, and LiteMol (Sehnal *et al*. 2017) introduced BinaryCIF (Sehnal *et al*. 2020) with downsampling to improve compression efficiency. Mol* (Sehnal *et al*. 2018) provides an open-source platform for molecular data delivery, analysis, and visualization. However, since these systems rely on simple compression or downsampling, they often introduce structural distortions (e.g. blurred atomic bond angles (Sehnal *et al*. 2017)), highlighting the need to balance compression efficiency and data fidelity in cryo-EM visualization.

## 3 Research overview

**Figure 2:**
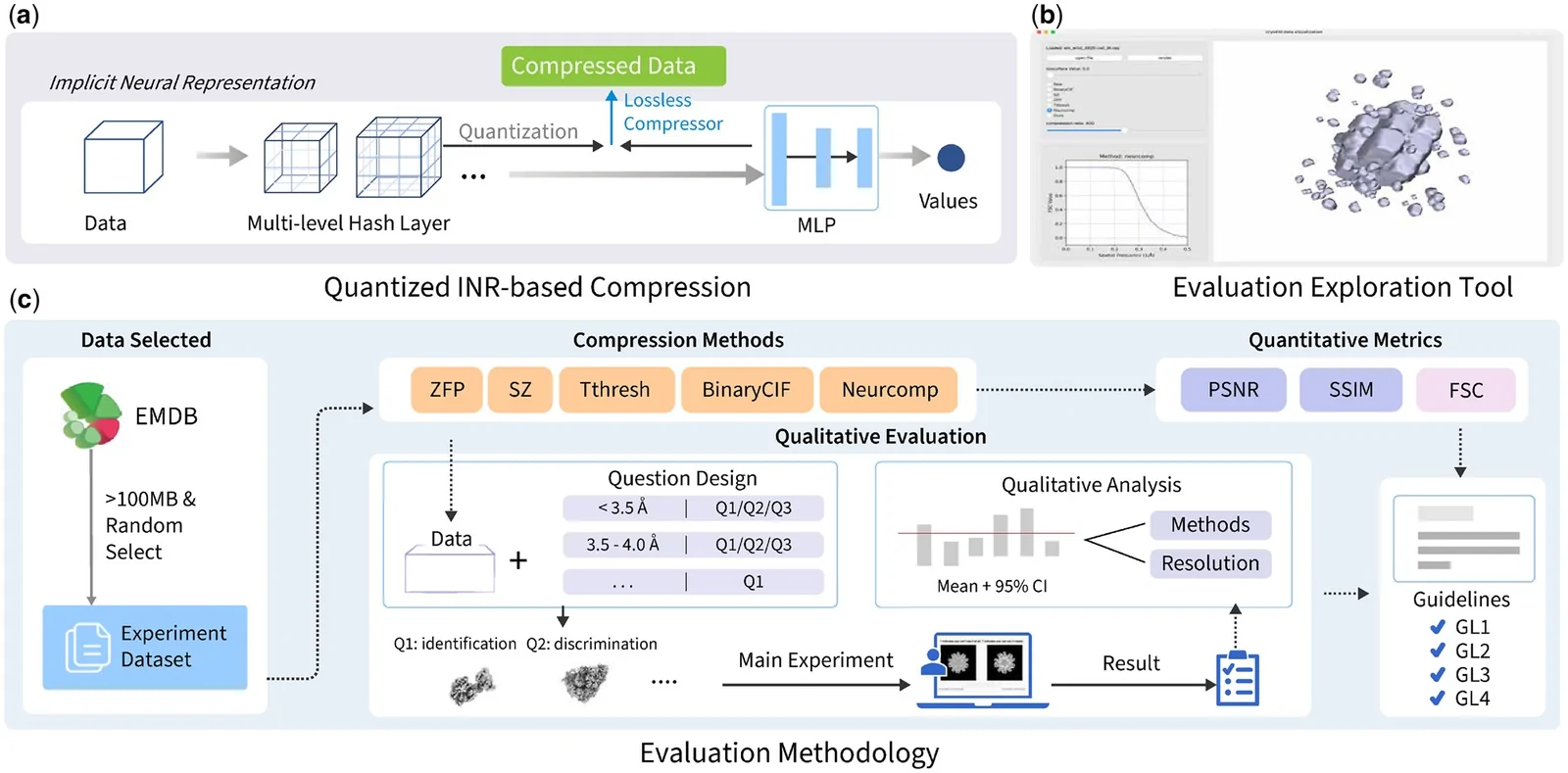
Research overview. We propose a hybrid compression pipeline that combines implicit neural representation with quantization (a). To assess its effectiveness, we design an evaluation framework incorporating cryo-EM datasets, compression methods, analytical tasks, and both quantitative and qualitative metrics (b). Finally, we introduce an interactive system for visualizing decompression results across different methods (c).

In this study, we focus on the core research question: *how can cryo-EM data be effectively compressed while preserving visual fidelity and supporting interactive analysis?* To address this, we propose a quantized implicit neural representation (INR) method that combines high compression efficiency with on-demand data access. To evaluate its effectiveness, we evaluate our approach against traditional and a classic INR-based compression method. We first describe our quantized INR-based compression approach (Section 4), followed by an introduction to our evaluation methodology (Section 5) and a detailed quantitative and qualitative experimental analysis of our evaluation (Section 6). Additionally, we summarize a set of guidelines for choosing compression methods in cryo-EM data visualizations, and provide an interactive evaluation exploration tool for users to access and explore our evaluation (Section 7). Figure 2 provides an overview of our research.

## 4 Quantized INR-based compression

**Figure 3:**
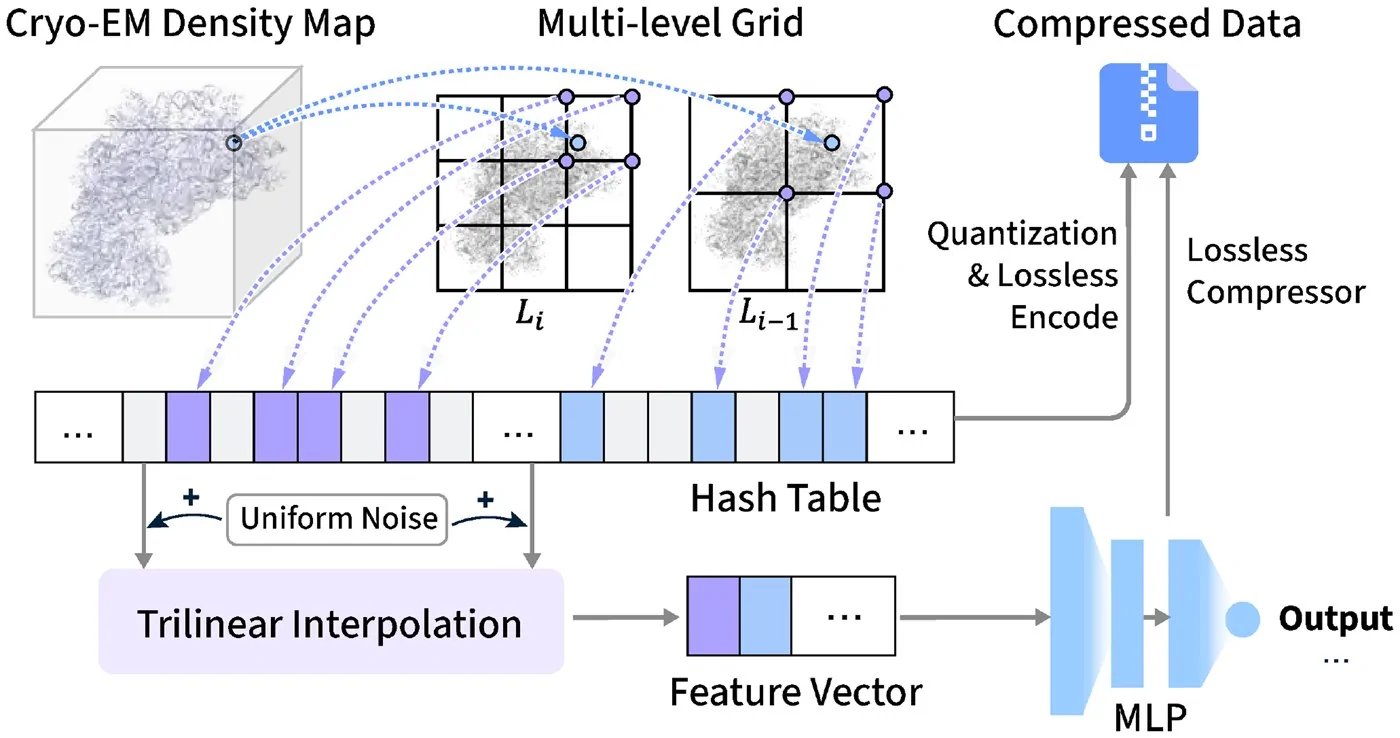
Quantized INR-based compression architecture. At each grid level, the feature vectors of the eight adjacent vertices corresponding to an input coordinate (four vertices per dimension are shown for simplicity) are stored in a hash table. The input feature vectors are obtained via trilinear interpolation and fed into a compact MLP to predict voxel densities. During training, uniform noise simulates quantization error. After training, the hash table is quantized and further compressed using lossless encoding.

We propose a quantized INR-based compression approach that leverages a multi-level hash grid (Section 4.1) to accelerate computation and incorporates quantization (Section 4.2) with a lossless compressor (Section 4.3) to reduce storage. Figure 3 illustrates the overall architecture.

### 4.1 Multi-level hash grid

We use a multi-level hash grid strategy (Müller *et al*. 2022) to accelerate computation while preserving details at multiple scales. Each input coordinate is encoded via feature vectors from surrounding grid vertices across different resolution levels, enabling efficient feature encoding and reconstruction. Specifically, we first embed the coordinates of a cryo-EM density map into a multi-level 3D grid $L:={\left\{L_{i}\right\}}_{i=1}^{N}$, where *N* is the number of grid levels, $L_{1}$ and $L_{N}$ correspond to the coarsest and finest grids, respectively. The resolutions of the grid levels, ${\left\{R_{i}\right\}}_{i=1}^{N}$, follow a geometric progression governed by a common ratio *b*, defined as:


(1) $$b=\text{exp}\;\left(\frac{\text{ln}R_{N}-\text{ln}R_{1}}{N-1}\right).$$

The resolution of the *i*-th level grid is determined by:


(2) $$R_{i}=R_{1}\cdot b^{i-1}.$$

The vertices in each grid level $L_{i}$ are stored in a hash table with a maximum hash table size $H_{\max}$. There are two possible cases regarding the relationship between the hash table size and the number of coordinate points: either the number of points is within the limit, or it surpasses it. When the number of coordinate points within the grid remains below $H_{\max}$, a bijective mapping between grid coordinates and hash table index is established using the following formula:


(3) $${\text{map}}_{i}(x,y,z)=x\cdot R_{i}^{2}+y\cdot R_{i}+z,$$

Where (x,y,z) denotes the 3D coordinates in grid space, $R_{i}$ is the grid resolution mentioned before. For the cases where the coordinate points surpass the maximum hash table capacity $H_{\max}$, the following hash function is used to compute the hash table index:


(4) $$\text{hash}(x,y,z)=(x\cdot p_{1}⊕y\cdot p_{2}⊕z\cdot p_{3})\;\text{mod}\;H_{\text{max}},$$

Where ⊕ represents XOR operation, (x,y,z) denotes the 3D coordinates in grid space, and $p_{1}$, $p_{2}$, and $p_{3}$ represent three distinct prime numbers. The modulo operation ensures that the resulting index falls within the valid range.

Hash collisions can occur when multiple grid coordinates map to the same hash table entry. Rather than using traditional conflict-resolution methods (e.g. chaining or open addressing) (Yusuf *et al*. 2021), our neural network treats the stored hash table values–feature vectors–as trainable parameters, implicitly resolving collisions through INR backpropagation. The feature vector dimension controls the amount of stored information: higher dimensions enable richer representations but increase storage requirements.

### 4.2 Quantization

Based on the multi-level hash grid representation, we further reduce storage by quantizing the learned feature vectors. Quantization introduces two challenges—non-differentiability and potential information loss—which we address by adding uniform noise during training (Ballé *et al*. 2017) to simulate quantization error. Combined with trilinear interpolation for smoothing, this enables the network to learn and compensate for quantization effects in an end-to-end manner. Specifically, we add uniform noise $U(-\frac{1}{2n},\frac{1}{2n})$ to the feature vectors, where *n* is the number of quantization levels. We set n=256 by default, corresponding to 8-bit quantization of 32-bit floats, balancing storage efficiency and fidelity. After training, we apply actual quantization to the hash table parameters:


(5) $$\hat{v}=\text{round}\left(\text{norm}(v)\cdot n-0.5n\right),$$

Where *v* denotes feature vector values across all hash tables, $\hat{v}$ represents the corresponding values after quantization, and *norm* refers to the min-max normalization.

### 4.3 Lossless compressor

After quantization, we further compress the serialized parameters using a lossless coding scheme to remove statistical redundancy. Our quantization strategy reduces storage and improves parameter compressibility, enabling more effective lossless compression. We apply LZMA2 (Pavlov 2024), which combines dictionary and range encoding, achieving an additional ∼1.5× reduction in network size. After lossy compression, embedding layers are quantized while MLP weights retain full precision. The parameters are then serialized into a platform-independent format and compressed with LZMA2 to remove statistical redundancies, ensuring lossless reconstruction.

### 4.4 Implementation details

We next describe the parameter settings and training strategy used in our implementation. We fix the minimum resolution at 16 and scale the maximum resolution to the largest input dimension in the multi-level grid. The grid has three hyperparameters: maximum hash table size ($H_{\text{max}}$), number of levels (*N*), and feature dimension per entry (*F*). We vary *N* from 8 to 16 with F=2 and find N=16 consistently performs best for most cryo-EM data, which we adopt. $H_{\text{max}}$ is automatically determined from the input size and target compression ratio, with grid parameters configured via geometric resolution progression and iterative adjustment to meet compression targets. Our model uses an MLP with two hidden layers and Leaky ReLU activations, with layer widths adapted to compression ratio (128 neurons below 400×, 64 neurons at 400× or higher). Training uses the Adam optimizer (Kingma and Ba 2017) ($\beta_{1}=0.9,\beta_{2}=0.99$) with MSE loss, sampling 32768 random coordinates per step and accelerated by *tiny-cuda-nn* (Müller 2021). Early stopping is applied when the average loss no longer improves.

## 5 Evaluation methodology

We propose an evaluation framework to evaluate our method against existing cryo-EM compression techniques, using datasets of varying resolutions and sizes, five domain-specific analytical tasks, three quantitative metrics, a qualitative visual assessment, and five candidate methods. The evaluation methodology is detailed in this section, with results presented in Section 6.

### 5.1 Domain-specific analytical tasks

Based on literature review (Blow 2002, Casañal *et al*. 2019) and consultations with three structural biology experts, we established a taxonomy of analytical tasks aligned with cryo-EM resolution levels. These tasks span from secondary structure identification at high resolution to macromolecular shape recognition at lower resolution, grouped into five categories: (i) <∼  *3.5 Å*: secondary structure (T1), large side chain and amino acid residues (T2), and side chain details (e.g. intermolecular interactions, ligand binding sites, and disulfide bonds) (T3); (ii) ∼  *3.5–4.0 Å*: T1, T3, side chains of amino acid residues (T4); (iii) ∼  *4.0–6.0 Å*: partial protein backbone (T5); (iv) ∼  *6.0–8.0 Å*: structural domain morphology (T6); (v) >∼  *8.0 Å*: overall protein topology (T7). Details on the analytical tasks, along with the number of cryo-EM datasets and trials, are provided in the supplementary material, available as supplementary data at *Bioinformatics* online.

### 5.2 Data collection

To evaluate compression methods across analytical tasks and resolution levels, we randomly selected 40 cryo-EM datasets from the open-access EMDB (wwPDB Consortium 2023) based on two criteria: (i) *Data size*–only volumes larger than 200 MB were included to ensure realistic compression needs and coverage of high-resolution data; (ii) *Spatial resolution*–datasets span from below 3.5 Å to above 8 Å. Table 1 summarizes the size and resolution distributions of the datasets.

**Table 1:** Size and resolution distribution of the datasets.

Resolution (Å)	< **3.5**	[3.5, 4)	[4, 6)	[6, 8)	≥ **8**
Number	13	6	6	4	11
**Size (GB)**	< **0.3**	**[0.3, 0.5)**	**[0.5, 0.7)**	**[0.7, 1)**	≥ **1**
Number	15	12	9	2	2

### 5.3 Candidate compression methods

We evaluated five open-source volumetric compression methods with different parameter settings and our proposed quantized neural representation approach (Section 4). Topology-preserving compressors were not included in this evaluation because their topology-specific objectives, parameter settings, and evaluation metrics are not directly aligned with our unified visualization-oriented protocol. To further examine this scope boundary, we provide a case in the supplementary material, available as supplementary data at *Bioinformatics* online, where TopoSZ (Yan *et al*. 2024) shows limited improvement in compression ratio, high compression time, and bridge-like artifacts on a noisy cryo-EM density map.

- *BinaryCIF* (Sehnal *et al*. 2020): a widely used baseline downsampling method. We used DensityServer (Sehnal *et al*. 2017) to convert data to.*bcif* format and CIFTools (Sehnal *et al*. 2020) to reconstruct 3D volumes. The default *quantization level* (float32 to int8) was used, while *maximum downsampling levels* (1–3) were varied to control compression.
- SZ (Di and Cappello 2016): it controls quality through an error bound rather than a target compression ratio. Lower error bounds improve quality but reduce compression ratios. We tested *relative-bound-ratio* of 0.01, 0.05, 0.1, 0.2, 0.4, 0.6, and 0.8.
- *Tthresh* (Ballester-Ripoll and Pajarola 2016): similar to SZ, we set the error bound to control compression quality using the same strategy.
- *ZFP* (Lindstrom 2014): using the fixed-precision mode of ZFP, the relative error is governed by a positive integer *x*, corresponding to the relative error bound $2^{-x}$. We tested values of *x* from 1 to 6.
- *Neurcomp* (Lu *et al*. 2021): it allows direct specification of the target *compression ratio*. We evaluated target compression ratios of 50, 100, 200, 400, 600, 800, and 1500 after the compression process.

Please refer to our supplementary material, available as supplementary data at *Bioinformatics* online for additional details on parameter settings. In our experiments, we explore a broad range of compression ratios, from moderate (50×) to aggressive (up to 1500×), to address practical challenges posed by the rapidly growing size of cryo-EM maps. This challenge is also reflected in the EMDB, where maps larger than 500 MB account for 12% of entries, and more than 1740 maps exceed 1 GB (wwPDB Consortium 2023), such as EMD-7315 (>2 GB).

### 5.4 Quantitative metrics

We evaluate all methods using three standard metrics: PSNR (Huynh-Thu and Ghanbari 2008) for data differences, SSIM (Wang *et al*. 2004) for visual similarity, and FSC for structural consistency between original and decompressed data. *PSNR* measures the ratio of maximum signal to reconstruction noise, with higher values indicating better fidelity. *SSIM* assesses similarity in luminance, contrast, and structural patterns, with values closer to 1 indicating stronger similarity. *FSC* (van Heel and Schatz 2005) is widely used in structural biology to evaluate structural similarity:


(6) $$\text{FSC}(\omega )=\frac{\sum_{\omega }F_{1}(\omega )\cdot F_{2}^{*}(\omega )}{\sqrt{\sum_{\omega }{\left|F_{1}(\omega )\right|}^{2}\cdot \sum_{\omega }{\left|F_{2}(\omega )\right|}^{2}}},$$

Where $F_{1}(\omega )$, $F_{2}(\omega )$ represent the Fourier spectrum functions of two sets of input cryo-EM data at frequency ω, and $F^{*}$ represents complex conjugate. The value range of FSC is [−1,1]. FSC→1 indicates a high similarity between the original cryo-EM data and the decompressed one. In our experiment, $F_{1}$ and $F_{2}$ indicate Fourier Transform of the ground truth and decompressed cryo-EM data.

### 5.5 User study

#### 5.5.1 Study design

We conducted a user study using a seven-point Likert scale with a one-factor mixed design (within-subject for compression method and between-subject for trial assignment) to evaluate perceived visual quality. The single factor was compression method, with six levels corresponding to five baselines and our approach. Participants rated the visual quality of decompressed data relative to the original on a scale from 1 (very poor) to 7 (excellent). Each participant evaluated all six methods over a representative subset of trials covering all analytical tasks, with method order randomized to reduce learning effects. The dependent variable was the quality score, and the independent variable was the compression method.

#### 5.5.2 Stimuli

To ensure fair comparison, the ideal setup would use identical compression ratios across methods. However, as discussed in Section 5.3, the four traditional volume compression methods do not allow precise ratio control. We therefore selected a target compression ratio ϵ that best balanced cross-method consistency and visual quality based on quantitative metrics, allowing a tolerance of ±50% when exact matching was not possible. Based on results in Section 6.2, we set ϵ=400 and retained 40 datasets whose compressed results fell within the ϵ ± 50% range for all methods. The corresponding data size and resolution distributions are reported in Table 1.

#### 5.5.3 Tasks

Participants were required to rate visual quality by comparing side-by-side videos, with the original data on the left and the decompressed result on the right. Isosurfaces were generated using Marching Cubes with EMDB-recommended thresholds and rendered from identical viewpoints with synchronized rotations to enable comparison across angles. Although the ground truth was always shown on the left, this did not introduce position bias, as it served only as a reference and the synchronized presentation ensured fair evaluation. To guide assessment, we aligned ratings with domain-specific analytical tasks by asking targeted questions such as ‘*Can you clearly see* <*task name*>?’, corresponding to the tasks in Section 5.1.

#### 5.5.4 Participants

We recruited participants via *Prolific*, targeting individuals with at least a Bachelor’s degree in biology, biochemistry, biomedical sciences, or related fields to ensure expertise in evaluating cryo-EM density maps. We selected 40 datasets at varying resolutions and paired them with relevant tasks, yielding 87 trials per compression method. Using a mixed design, each participant evaluated all six methods, with trials randomly divided into five groups to balance workload. Based on an expected effect size of 0.8 and power of 0.8, at least 15 participants per group were required. In total, 75 participants were recruited (37 males, 34 females, 4 undisclosed) and compensated about $6.80 each for an average completion time of 35 min.

#### 5.5.5 Procedure

The experiment consisted of three phases. In the training phase, participants viewed videos to become familiar with the tasks and interface; responses were not recorded. In the evaluation phase, they compared original and reconstructed data in side-by-side videos and rated restoration quality on a seven-point Likert scale, focusing on overall outline (low resolution) or local details (high resolution), with 1 indicating unrecognizable structures and 7 indicating exceptional clarity. Finally, in the demographic phase, anonymized participant information was collected to analyze population distribution.

## 6 Experimental results

We implemented our quantized INR-based compression method in PyTorch and trained it on a system with an Intel Xeon Platinum 8352 V CPU (12 cores, 2.1 GHz) and an NVIDIA RTX 4090 GPU (24 GB VRAM). All candidate methods were tested in the same environment. We evaluated our approach against five alternatives through qualitative evaluation, quantitative analysis, and user study.

### 6.1 Qualitative comparisons

**Figure 4:**
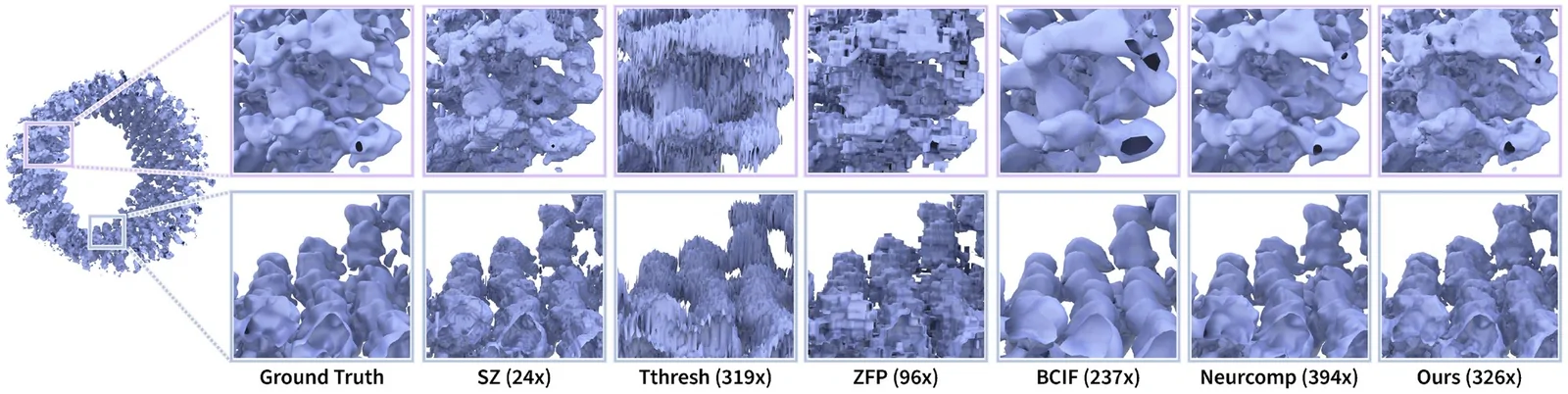
Qualitative evaluation. We present a highlighted local region of the EMD-3075 data (189 MB, 8.0 Å), which was compressed by the six compression methods at ϵ≈300, together with the ground truth. Even at lower ratios (ϵ<100), SZ and ZFP exhibit inferior visual fidelity compared to Neurcomp and ours at higher ratios (ϵ>300). At similar ratios (ϵ≈300), Neurcomp and Ours maintain superior detail preservation and overall quality over Tthresh.

Figure 4 shows qualitative comparisons of a cryo-EM dataset compressed by different methods against their ground truth versions, which shows Cyclophilin A in complex with the assembled HIV-1 capsid (Electron Microscopy Data Bank entry, EMD-3075 (Liu *et al*. 2016), 189 MB, 8.0 Å) compressed at comparable ratios using different methods. In this example, *Neurcomp and our method both produce high-quality reconstructions*. Tthresh generally retains detail but causes distortions on complex structures, while BCIF yields smooth surfaces at the cost of substantial structural loss due to downsampling.

### 6.2 Quantitative comparisons

We conducted four quantitative analyses: rate–distortion performance, FSC evaluation, computational efficiency, and an ablation study of our quantization operator.

#### 6.2.1 Rate-distortion performance

We conducted a rate–distortion analysis of the six methods to evaluate the trade-off between compression ratio and reconstruction quality. Each dataset (Section 5.2) was compressed under parameter settings (Section 5.3) to generate a range of ratios. For methods without explicit ratio control, we defined the ratio as the size of the original data divided by the compressed size under a given setting. Distortion was measured using PSNR and SSIM, and aggregated across datasets as a weighted average with original data size as the weight.

**Figure 5:**
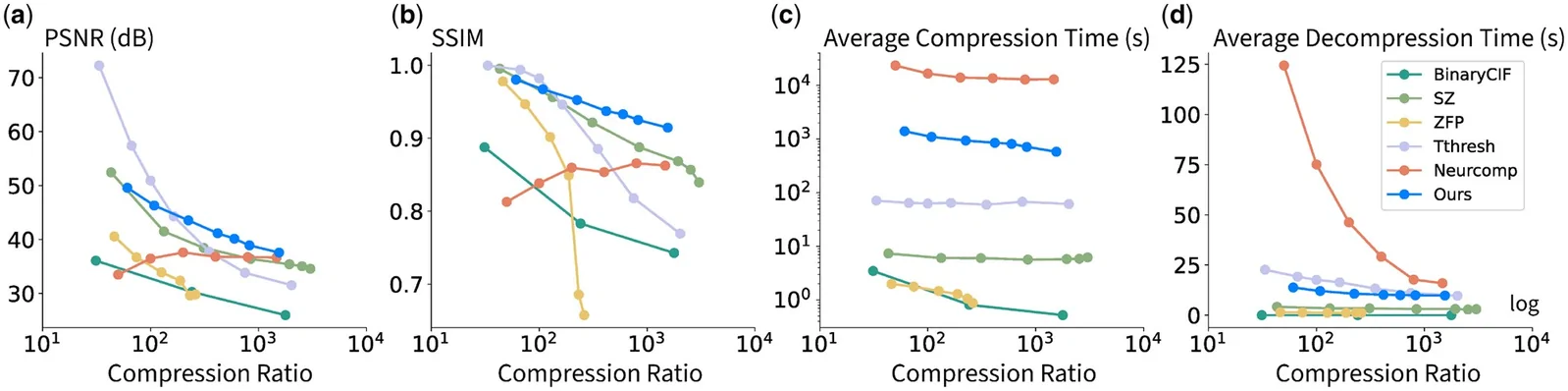
Quantitative evaluation. We present four curves that measure the quantitative metrics across compression ratios for the six candidate methods based on 40 datasets (>200 MB): (a) PSNR, (b) SSIM, (c) average compression time, and (d) average decompression time. Each method is consistently color-coded across the plots. Compression ratios are plotted on a logarithmic x-axis spanning from 10 to $10^{4}$.

Figure 5a and b show the rate–distortion curves, measured by PSNR and SSIM. While Tthresh performs best at lower compression ratios (below 400×), *our method clearly outperforms all others at higher ratios*. Neurcomp shows slight improvement between 50× and 200×, likely because noise in cryo-EM data hinders its convergence at lower ratios due to its large parameter count. By incorporating quantization and a multi-level grid into INR optimization, *our method overcomes this limitation and achieves performance comparable to or better than SZ*. Figure 5a further demonstrates that for PSNR values below 40, *our method achieves higher compression ratios than nearly all other methods*.

#### 6.2.2 Computational efficiency

We measured compression and decompression times for all six methods. Compression time was recorded from method initialization to the generation of the compressed output, while decompression time was measured from input of the compressed data to the production of the reconstructed volume. To ensure a fair comparison, we computed weighted averages across datasets. Figure 5c and d show the results: although INR-based methods generally require longer compression, *our method is roughly ten times faster than Neurcomp*, and its *decompression speed surpasses even Tthresh*, while remaining comparable to traditional methods. This efficiency is achieved through our multi-level hash grid architecture and early-stopping strategy.

#### 6.2.3 FSC analysis

**Figure 6:**
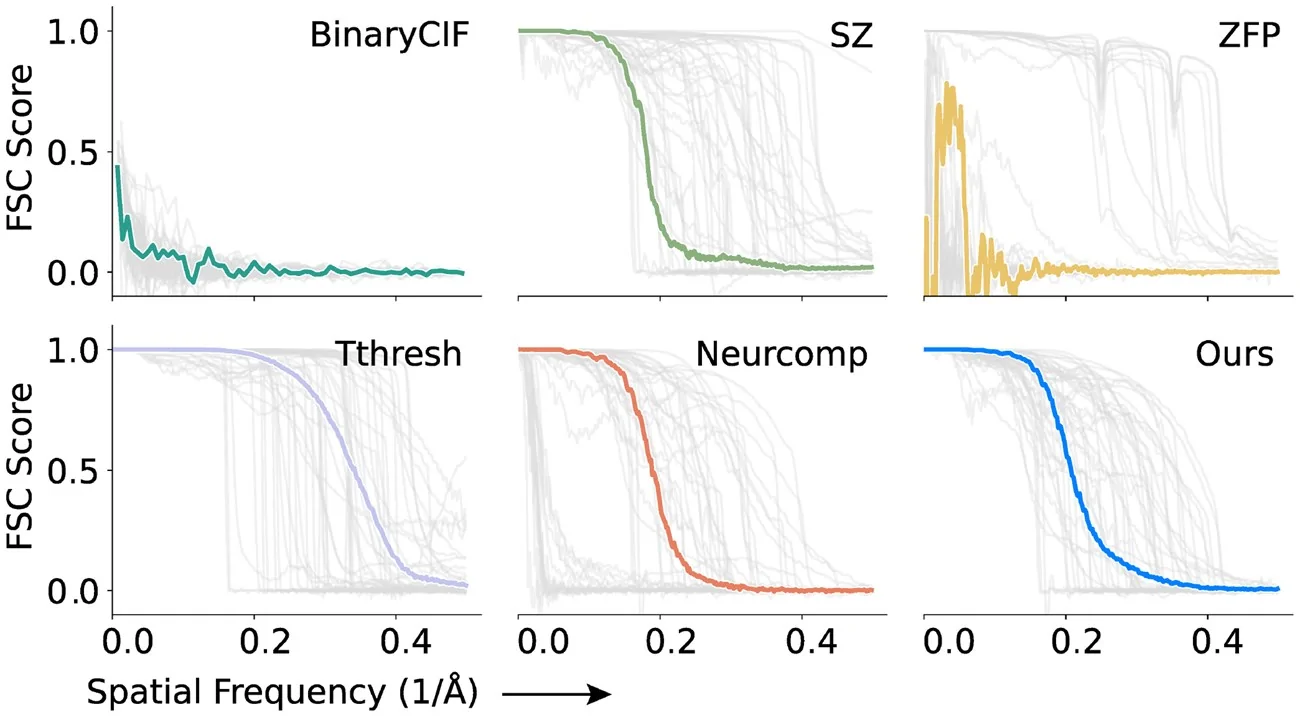
FSC result. We evaluated the six compression methods using FSC on the selected user study data. Each curve shows structural similarity across spatial frequencies, reflecting how well a method preserves structural information. The *x*-axis shows spatial frequency (1/Å), and the *y*-axis shows FSC scores. Individual curves correspond to data-specific FSC results for each method. For clarity, highlighted curves denote one representative dataset, while faded curves indicate the remaining datasets.

We performed FSC analysis on the user study dataset, which was maintained at an approximately consistent compression ratio of ϵ=400 (Section 5.5). Figure 6 shows FSC results for all methods, highlighting EMD-9249 (669.92 MB, 7.7 Å) while fading others. The *x*-axis denotes spatial frequency (inverse spatial resolution) from low to high, and the *y*-axis shows FSC values (Eq. 6) between compressed and original data. An effective method should preserve stable FSC at low frequencies without sharp drops at higher frequencies.

Figure 6 shows that most methods closely match the original data at low frequencies, with similarity decreasing at higher frequencies. Tthresh performs best on the highlighted dataset, with noticeable degradation only when 1/Å>0.4. In contrast, BCIF shows the poorest FSC across all frequencies, likely due to aggressive downsampling, while ZFP exhibits strong low-frequency fluctuations, indicating instability. Compared with other methods, our approach produces a smoother FSC curve and preserves higher similarity than both SZ and Neurcomp, especially in medium-to-high frequency regions.

#### 6.2.4 Ablation study

We conducted an ablation study to evaluate the effectiveness of our quantization strategy. We randomly selected 20 additional datasets from EMDB (wwPDB Consortium 2023), distinct from those used in our evaluation. These datasets were compressed at three different compression ratios: 200, 400, and 800, both with and without the quantization strategy.

**Figure 7:**
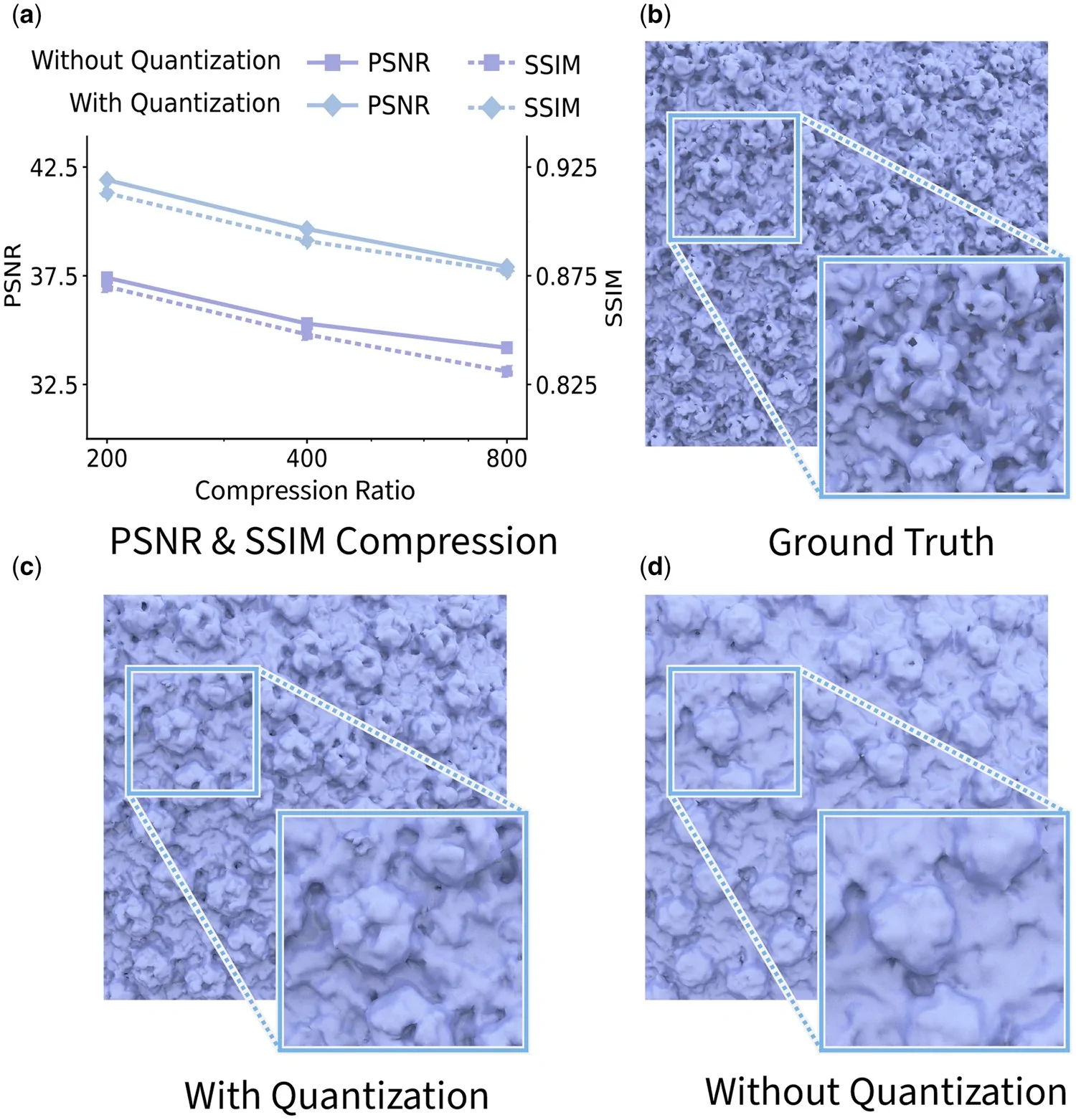
Ablation study results. The EMD-8484 dataset (334.1 MB, 15.0 Å) is compressed at a ratio of 400 using our method, with and without quantization. Panels (a)–(d) show: (a) PSNR and SSIM of the two strategies across three compression ratios, (b) the original data, (c) the compressed result with quantization, and (d) the compressed result without quantization.

We examined quantitative metrics such as PSNR and SSIM, as shown in Fig. 7a. Our method with quantization consistently outperforms the non-quantized version across all tested compression ratios. The performance of the quantized approach at ϵ=800 is *comparable to the non-quantized method at a* 4×  *lower compression ratio*, highlighting its effectiveness in maintaining visual quality at high compression levels. For a detailed analysis, we present a comparison of our compression method with and without quantization at a compression ratio of 400, as shown in Fig. 7c and d. While both strategies lose some detail at a high compression ratio compared to the ground truth (Fig. 7b), *the strategy with quantization preserves significantly more structural details*.

#### 6.2.5 On-demand access evaluation

The key advantage of INR-based compression is on-demand access, which enables interactive exploration without full decompression and significantly reduces memory usage during rendering. For example, in ray-casting–based volume rendering, only samples along ray paths are decompressed, avoiding loading the entire volume into VRAM (Wu *et al*. 2023).

**Figure 8:**
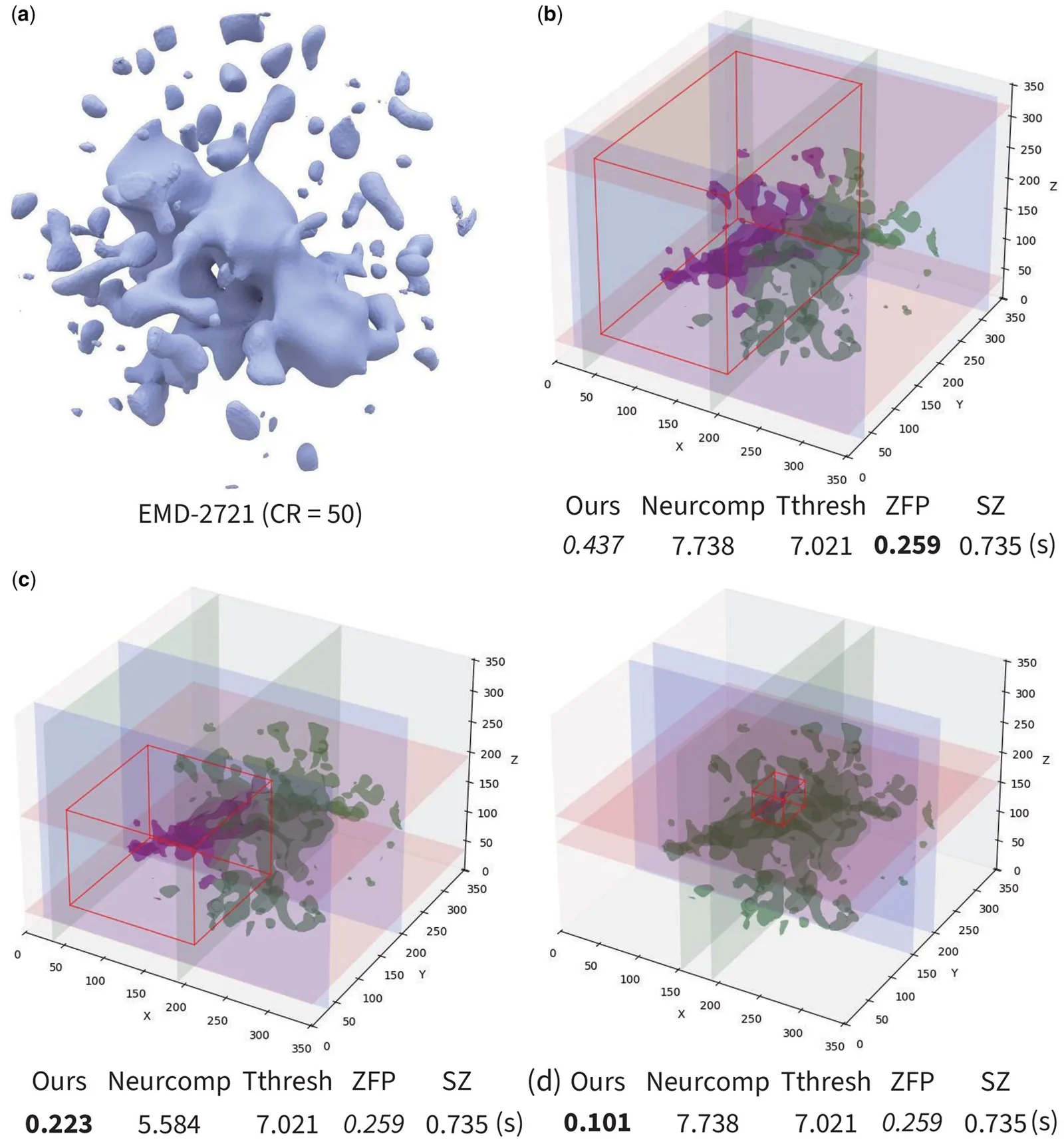
On-demand access. We demonstrate the on-demand access capability on the EMD-2721 dataset (166.38 MB, 66.0 Å). Panels (b)–(d) show progressively narrower user-selected ranges with correspondingly reduced decompression time. For methods without on-demand access, we also report decompression time for the full volume. The fastest and second fastest times in each group are highlighted in bold and *italic*, respectively.

We evaluated the memory efficiency and on-demand decompression performance of our method. First, we assessed memory consumption during decompression using a 6.43 GB cryo-EM dataset compressed at a ratio of 1500. Decompressing a 1/8 data block consumed 705.6 MB RAM and 1.6 GB VRAM, while a 1/64 block used 705.8 MB RAM and 883 MB VRAM—both substantially lower than the original data size. We next measured the runtime for these decompressions: a 1/8 block required 24.44 s, and a 1/64 block required 8.08 s. To further demonstrate on-demand access for interactive visualization, we generated data slices across different ranges, as illustrated in Fig. 8. The results show that our method achieves the lowest decompression time among all alternative methods when the user-selected region is small, confirming that *our method efficiently supports on-demand access* while maintaining minimal memory usage.

### 6.3 User study

**Figure 9:**
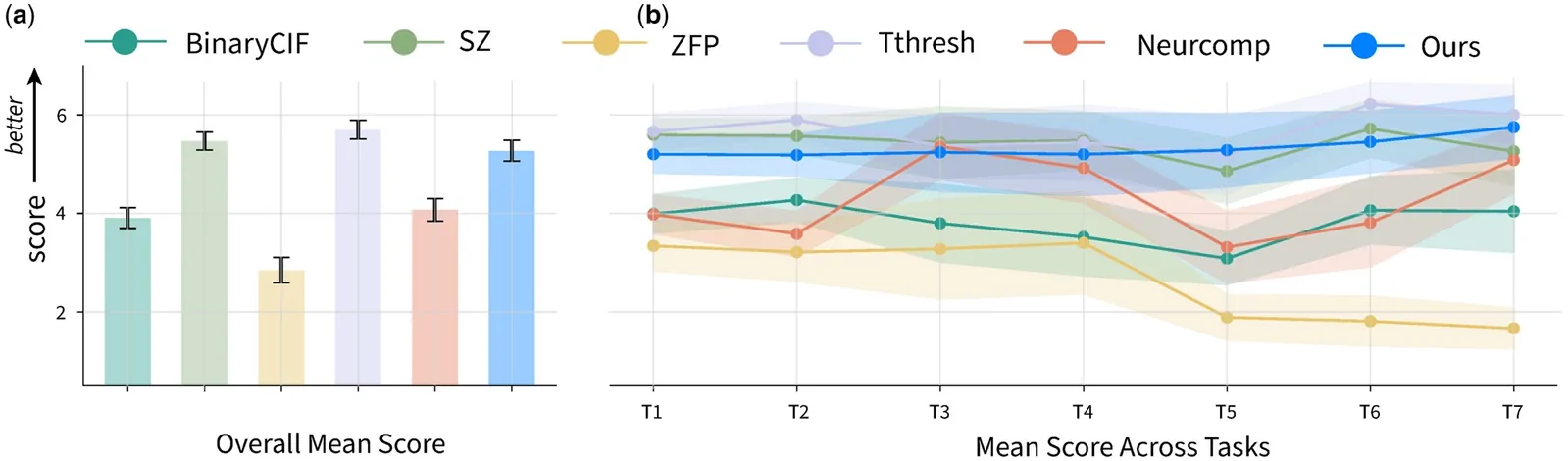
User study results. We report the mean scores along with their 95% confidence intervals. (a) shows the overall mean scores for the six compression methods, while (b) presents each method’s performance across different tasks.

We conducted the user study on Prolific following the methodology described in Section 5.5. We collected 41 valid responses from 75 participants. As shown in Fig. 9a, we report the mean scores with 95% confidence intervals for the six compression methods. Overall, our method outperforms Neurcomp but scores slightly lower than the top-performing traditional methods, Tthresh and SZ.

Figure 9b presents the mean scores aggregated by analytical tasks (Section 5.1). Among all methods, our method, Tthresh, and SZ clearly outperform the others across all tasks. Tthresh achieves the highest or near-highest scores, while our method demonstrates strong and balanced performance: *surpassing Tthresh and SZ* in the main-chain determination task *(T5)*, surpassing SZ in side-chain determination tasks (T4), matching SZ on general structure pattern tasks (T5, T7), and remaining highly competitive overall. In contrast, ZFP and BinaryCIF consistently yield the lowest scores across both overall evaluations and resolution levels. These results underscore that our quantized multi-layer grid strategy effectively preserves structural fidelity and balances visual quality across analytical tasks.

## 7 Guidelines and tool

### 7.1 Guidelines

Based on our comprehensive study of various compression methods for cryo-EM data, we propose four guidelines to assist domain experts, software designers, and visualization practitioners in selecting suitable compression strategies for cryo-EM visualization:

*G1. Balance between compression ratio and visual quality*. Tthresh performs best at low compression ratios (<400×), preserving fine details but introducing artifacts at higher ratios. In contrast, INR-based methods support higher compression while retaining overall structure. Ours stands out at high compression ratios, maintaining both fidelity and robustness where traditional methods degrade.

*G2. Time efficiency for interactive use*. Traditional methods are generally faster than INR-based ones, with BinaryCIF and ZFP offering the highest speed but lower compression and visual quality. Our method substantially improves INR efficiency—around ten times faster than Neurcomp—while maintaining reasonable visual quality, making it suitable for interactive scenarios such as large-scale cryo-EM visualization on mobile or memory-constrained devices.

*G3. On-demand access for large-scale data*. While most traditional methods require full decompression, INR-based approaches enable selective access. Ours further enhances this by combining efficient memory usage with on-demand decompression, making it effective for large-scale cryo-EM data in constrained environments.

*G4. Matching compression to analytical tasks*. At low compression ratios, Tthresh performs best across analytical tasks. At higher ratios, SZ is more effective below 6.0 Å, whereas our method performs best above 8.0 Å, especially in preserving chain structures and overall morphology.

### 7.2 Evaluation exploration tool

**Figure 10:**
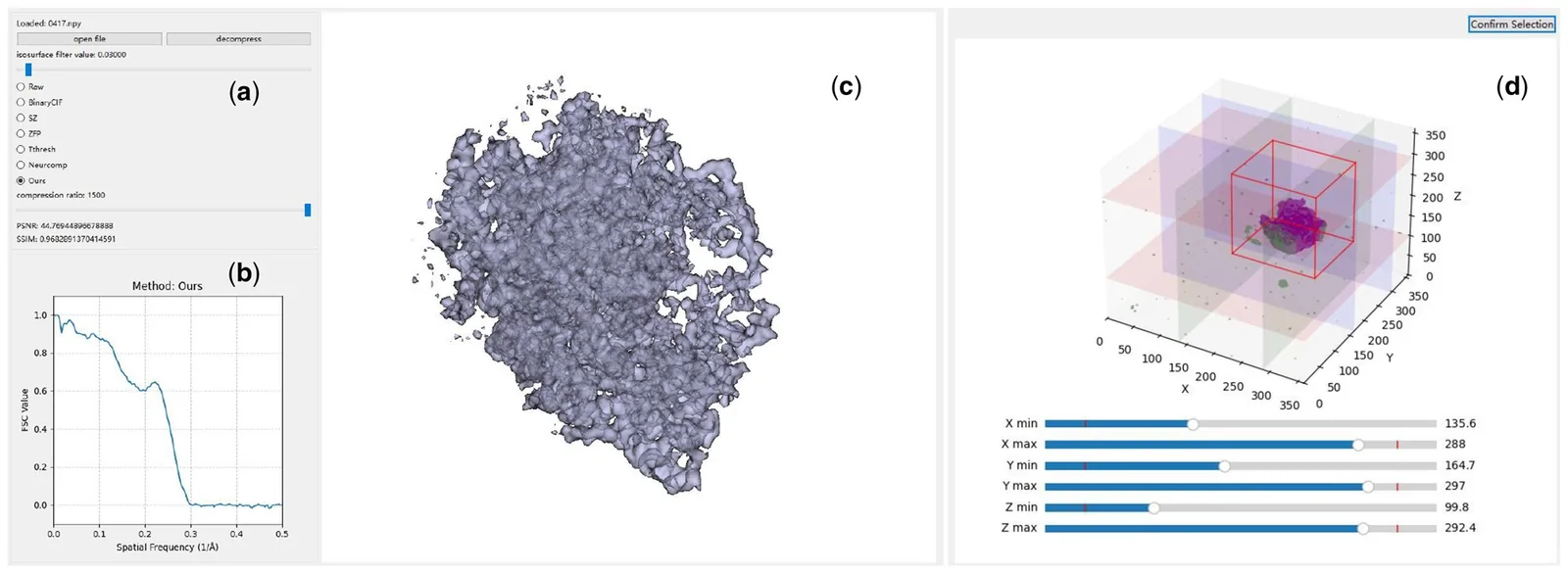
Evaluation exploration tool. Users load data and select a compression method in (a), then view reconstructed isosurfaces in (c), updated metrics in (a), and FSC curves in (b). When our method is selected, the decompression tool in (d) supports region-based decompression and updates the visualization in (c).

To support profiling, analysis, and comparison of data compressed by different methods, we developed an interactive web-based tool that integrates FSC curve visualization with automated quality metric computation. The system also offers region-based local decompression and user-controlled iso-surface generation for analysis at specific level sets. As shown in Fig. 10, the system consists of two main components: (i) an interactive cryo-EM visualization interface with a control panel, FSC plotting area, and 3D isosurface viewer; and (ii) a decompression region selector with interactive selection and confirmation. Its core functions include isosurface extraction, data selection, compression method assignment, and parameter configuration.

## 8 Conclusion

In this paper, we introduced a quantized INR-based compression method for cryo-EM data, combining multi-level hash grids and quantization to support high compression ratios, on-demand access, and interactive visualization. We evaluated it against traditional and INR-based baselines using quantitative metrics, analytical tasks, and a user study, and developed an exploration tool with practical guidelines. Results show that our method offers a strong trade-off among storage efficiency, visual fidelity, and computational performance.

### 8.1 Limitations and future work

Our study has several limitations that suggest future directions. We did not evaluate topology-preserving methods (e.g. contour trees (Yan *et al*. 2024)). Although these methods are valuable for preserving structural features, their topology-specific objectives, runtime costs, and visual behavior are not directly aligned with our visualization-oriented evaluation, which emphasizes compression efficiency, visual fidelity, and interactive on-demand access. Extending future benchmarks to include topology-preserving methods would enable a broader assessment of analytical fidelity. Our analysis relied on general metrics; incorporating domain-specific measures such as RSCC (Zhang *et al*. 2025) in collaboration with structural biologists would yield deeper insights. The evaluation was limited to cryo-EM density maps with resolutions of ≥1.8 Å; extending to higher-resolution and larger datasets will further validate robustness. While we focused on iso-surface visualization due to its prevalence in structural biology, applying our method to more general volume data and addressing compression of raw cryo-EM images are important for future work.

## Supplementary Material

btag547_Supplementary_Data
