# Supplementary material for: Quantized neural representation for lossy cryo-EM compression
Source: Bioinformatics. 2026 Jul 23;42(8):btag547. doi: 10.1093/bioinformatics/btag547 (PMC13470431; doi:10.1093/bioinformatics/btag547)
Supplement: Supplementary file 1 [file btag547_supplementary_data.pdf]

Quantized Neural Representation for Lossy Cryo-EM Compression (Supplementary Materials)

Abstract—This supplemental material provides comprehensive experimental details and results that complement our main paper. We present: (1) Some specific details of the user study. (2) Brief information about all the data used in the experiment. (3) Some hyperparameter setting. (4) Further analysis on CPU & GPU occupancy. More detailed experimental data and the source code of our exploration tool and quantized neural representation method are available at `https://doi.org/10.5281/zenodo.19688284`, which includes the release version and a link to the GitHub repository.

1 User Study

Section 5.5 in the main paper introduces the methodology of our user study. In this section, we provide a more detailed explanation of the task settings, including the process of determining appropriate thresholds for each decompressed Cryo-EM data and how each specific trial was designed in accordance with domain-specific tasks.

1.1 Threshold calculation method

To compare visualization effects, we need to extract the same iso-surface from different decompression results of the same data and render them. However, due to the changes in the value distribution range after decompression, using the same threshold for all compression results of the same data can lead to inconsistent iso-surfaces. To ensure the consistency of the iso-surfaces, we apply the following method to adjust the threshold of the decompressed data.First, we extract iso-surface from the original data based on the recommended threshold values provided by EMDB [8]. Next, for each vertex coordinate in the iso-surface, we locate its corresponding density value in the decompressed data (using trilinear interpolation if it does not lie on a voxel grid point). Finally, we calculate the average of all obtained density values to derive the corresponding iso-surface threshold value for the decompressed data.

1.2 Trial settings

We have established a taxonomy of analytical tasks corresponding to different resolution levels. Meanwhile, the 40 selected protein data have different resolutions and thus fall into different resolution levels. We combine the tasks with the proteins within each resolution levels, resulting in a total of 53 trials. The specific details are listed in the Tab.1.

Resolution Levels	Specific questions	Task Num.	Protein Data Num.	Trial Num.
< 3.5 Å	Compared with the left image, can the secondary structures of the protein (such as α-helices and β-sheets) be clearly seen in the right image ?	3	13	24
	Compared with the left image, can the shapes of the larger side chains and some amino acid residues in the protein be clearly seen in the right image ?			
	Compared with the left image, can the intermolecular interactions of the protein, ligand binding sites, disulfide bonds and other structures be clearly seen in the right image ?			
3.5 - 4.0 Å	Compared with the left image, can the secondary structures of the protein (such as α-helices and β-sheets) be clearly seen in the right image ?	3	6	18
	Compared with the left image, can the side chains of the amino acid residues in the protein be clearly seen in the right image ?			
	Compared with the left image, can the intermolecular interactions of the protein, ligand binding sites, disulfide bonds and other structures be clearly seen in the right image ?			
4.0 - 6.0 Å	Compared with the left image, can part of the main chain of the protein (such as the α chain) be clearly seen in the right image ?	1	6	4
6.0 - 8.0 Å	Compared with the left image, can the structural domain morphology be clearly seen in the right image ?	1	4	4
> 8.0 Å	Compared with the left image, can the overall shape of the protein (general outline, main features) be clearly seen in the right image ?	1	11	3
Total	-	9	40	53

Table 1: Trials distribution across different resolution levels.

2 DATASET

2.1 Evaluation

In the evaluation, we randomly selected 40 data larger than 200MB from the first 10,000 entries in EMDB, as shown in Tab.2.

Name	Size (MB)	Resolution (Å)	Name	Size (MB)	Resolution (Å)	Name	Size (MB)	Resolution (Å)
EMD-1537	560.97	30.0	EMD-9733	347.61	25.0	EMD-0264	476.84	4.6
EMD-4365	669.92	10.4	EMD-0402	216.00	3.6	EMD-1939	320.54	8.9
EMD-7315	1953.13	7.8	EMD-6038	1423.83	6.0	EMD-5147	476.84	7.0
EMD-4129	421.88	3.06	EMD-6817	244.14	3.46	EMD-9249	669.92	7.7
EMD-4132	282.62	3.75	EMD-7593	216.00	3.25	EMD-9529	536.38	4.2
EMD-8130	465.48	5.8	EMD-5829	307.55	8.3	EMD-8391	244.14	4.4
EMD-9672	324.95	2.84	EMD-7769	512.00	3.2	EMD-2650	282.62	3.4
EMD-2649	262.91	3.35	EMD-9309	512.00	3.0	EMD-2142	729.00	4.5
EMD-8544	244.14	17.0	EMD-0352	512.00	3.21	EMD-1121	327.17	9.0
EMD-4323	216.00	4.9	EMD-3305	216.00	8.7	EMD-5993	515.01	12.6
EMD-9194	244.14	2.83	EMD-6637	244.14	3.9	EMD-6639	216.00	14.5
EMD-6896	421.88	3.3	EMD-3825	244.14	14.0	EMD-5233	781.25	3.9
EMD-6771	421.88	3.4	EMD-3574	620.92	3.1	EMD-6775	421.88	3.9
EMD-6270	282.62	3.5						

Table 2: Details of the 40 data used for evaluation.

2.2 Ablation Study

In our ablation study, we randomly selected 20 new data from EMDB using the same criterion as before, distinct from those used in our evaluation, as shown in Tab.3.

Name	Size (MB)	Resolution (Å)	Name	Size (MB)	Resolution (Å)	Name	Size (MB)	Resolution (Å)
EMD-0029	244.14	22.0	EMD-4115	137.09	3.2	EMD-7988	216.00	4.33
EMD-0548	216.00	6.5	EMD-4351	177.98	4.5	EMD-8320	512.00	3.5
EMD-1375	244.14	7.9	EMD-5184	188.56	17.6	EMD-8334	177.98	4.4
EMD-1648	307.55	7.8	EMD-5774	163.56	20.0	EMD-8484	828.10	15.0
EMD-2461	103.00	53.42	EMD-7062	125.00	3.7	EMD-8622	125.00	3.7
EMD-2932	244.14	14.9	EMD-7589	1423.83	2.73	EMD-8951	421.88	4.16
EMD-4059	266.78	3.9	EMD-7982	216.00	3.26			

Table 3: Details of the 20 data used for ablation study.

3 HYPERPARAMETER SETTING

We tested three groups of MLP neuron configuration introduced in Sec.4.4 with respect to relative quantitative metrics as shown in Tab. 4, which supports our hyperparameter setting selection.

4 FURTHER ANALYSIS ON CPU & GPU OCCUPANCY

In our experiments, the SZ [4], ZFP [3,5], and BinaryCIF [7] were executed on a single-core of CPU. Despite this, these three methods achieved speeds significantly faster than other approaches. Tthresh [1,2] was run on a multi-core CPU (12 cores); however, its performance was noticeably slower compared to other non-deep learning methods. Both Neurcomp [6] and our proposed method were executed on a GPU, but these two methods also consumed the most time.

In our experiments, we did not record detailed GPU utilization data. However, based on our observations, neither neural-network-based method fully leveraged GPU resources. GPU utilization ranged between 40% and 80%, varying with the neural network size, which was influenced by the original data size and target compression ratio. This finding suggests that both methods possess untapped potential for scaling to larger datasets, as increased network size typically leads to higher GPU occupancy.

Compression Rate	Neuron Num	Compression Time (s)	PSNR (dB)	SSIM
1500	32	566.1	41.6	0.993
	64	462.8	53.5	0.996
	128	510.7	47.0	0.991
600	32	500.4	50.6	0.998
	64	463.9	56.7	0.998
	128	470.9	50.2	0.998
300	32	476.1	59.2	0.999
	64	476.4	58.9	0.999
	128	527.0	57.8	0.999

Table 4: Quantitative metrics under different MLP neuron configurations.

4.1 Supplementary Evaluation of Topology-Preserving Compression

As a case, we tested TopoSZ on EMD-2721 (166.38 MB) to examine whether a topology-preserving compressor fits our cryo-EM visualization evaluation. With TopoSZ [9], increasing the global error bound ξ from 0.01 to 0.2 only moderately improves the compression ratio from 8.12 to 10.59, while requiring $179.7 \sim 439.1$ minutes. In this noisy density map, some fragmented components are also retained or connected into bridge-like structures under larger error bounds, which can be misleading for visual inspection. This case supports our decision not to include topology-preserving compressors in the main comparison, as their topology-specific objectives, runtime cost, and visual behavior are not directly aligned with our evaluation focus on compression ratio, visual fidelity, decompression efficiency, and on-demand access.

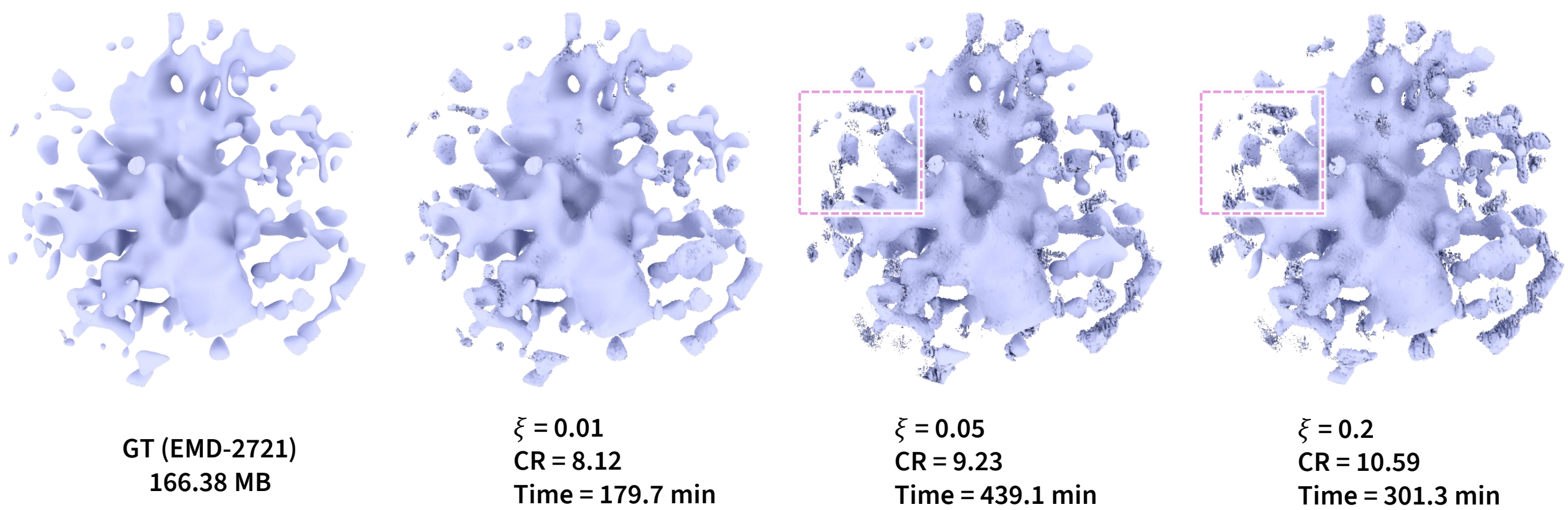

Fig. 1: TopoSZ was tested on EMD-2721 (166.38 MB) with varying global error bounds ξ. Increasing ξ only moderately improves the compression ratio while requiring long compression times. The highlighted regions show bridge-like structures formed by connected noisy density components under larger error bounds.

References

[1] R. Ballester-Ripoll, P. Lindstrom, and R. Pajarola. Tthresh: Tensor compression for multidimensional visual data. *IEEE Transactions on Visualization and Computer Graphics*, 26(9):2891–2903, 2020. doi: 10.1109/TVCG.2019.2904063 2

[2] R. Ballester-Ripoll and R. Pajarola. Lossy volume compression using Tucker truncation and thresholding. *The Visual Computer*, 32(11):1433–1446, November 2016. doi: 10.1007/s00371-015-1130-y 2

[3] J. Diffenderfer, A. Fox, J. Hittinger, G. Sanders, and P. Lindstrom. Error analysis of zfp compression for floating-point data. 05 2018. doi: 10.48550/arXiv.1805.00546 2

[4] X. Liang, S. Di, D. Tao, S. Li, S. Li, H. Guo, Z. Chen, and F. Cappello. Error-controlled lossy compression optimized for high compression ratios of scientific datasets. In *2018 IEEE International Conference on Big Data (Big Data)*, pp. 438–447, 2018. doi: 10.1109/BigData.2018.8622520 2

[5] P. Lindstrom. Fixed-rate compressed floating-point arrays. *IEEE Transactions on Visualization and Computer Graphics*, 20(12):2674–2683, 2014. doi: 10.1109/TVCG.2014.2346458 2

[6] Y. Lu, K. Jiang, J. A. Levine, and M. Berger. Compressive neural representations of volumetric scalar fields. *Computer Graphics Forum*, 40(3):135–146, 2021. doi: 10.1111/cgf.14295 2

[7] D. Sehnal, S. Bittrich, S. Velankar, J. Koča, R. Svobodová, S. K. Burley, and A. S. Rose. Binarycif and ciftools—lightweight, efficient and extensible macromolecular data management. *PLoS computational biology*, 16(10):e1008247, 2020. doi: 10.1371/journal.pcbi.1008247 2

[8] T. wwPDB Consortium. Emdb—the electron microscopy data bank. *Nucleic Acids Research*, 52(D1):D456–D465, 11 2023. doi: 10.1093/nar/gkad1019 1

[9] L. Yan, X. Liang, H. Guo, and B. Wang. Toposz: Preserving topology in error-bounded lossy compression. *IEEE Transactions on Visualization and Computer Graphics*, 30(1):1302–1312, 2024. doi: 10.1109/TVCG.2023.3326920 3
